# SmartSpectrometer—Embedded Optical Spectroscopy for Applications in Agriculture and Industry

**DOI:** 10.3390/s21134476

**Published:** 2021-06-30

**Authors:** Julius Krause, Heinrich Grüger, Lucie Gebauer, Xiaorong Zheng, Jens Knobbe, Tino Pügner, Anna Kicherer, Robin Gruna, Thomas Längle, Jürgen Beyerer

**Affiliations:** 1 Fraunhofer IOSB, Karlsruhe, Institute of Optronics, System Technologies and Image Exploitation, 76131 Karlsruhe, Germany; julius.krause@iosb.fraunhofer.de (J.K.); robin.gruna@iosb.fraunhofer.de (R.G.); thomas.laengle@iosb.fraunhofer.de (T.L.); 2 Fraunhofer IPMS, Institute for Photonic Microsystems, 01109 Dresden, Germany; heinrich.grueger@ipms.fraunhofer.de (H.G.); jens.knobbe@ipms.fraunhofer.de (J.K.); tino.puegner@ipms.fraunhofer.de (T.P.); 3 Julius Kühn-Institut, Institute for Grapevine Breeding Geilweilerhof, 76833 Siebeldingen, Germany; lucie.gebauer@julius-kuehn.de (L.G.); xiaorong.zheng@julius-kuehn.de (X.Z.); anna.kicherer@julius-kuehn.de (A.K.); 4 Vision and Fusion Laboratory (IES), Karlsruhe Institute of Technology (KIT), 76131 Karlsruhe, Germany

**Keywords:** industrial internet of things, near-infrared spectroscopy, miniaturized optical spectrometer, machine learning, smart viticulture

## Abstract

The ongoing digitization of industry and agriculture can benefit significantly from optical spectroscopy. In many cases, optical spectroscopy enables the estimation of properties such as substance concentrations and compositions. Spectral data can be acquired and evaluated in real time, and the results can be integrated directly into process and automation units, saving resources and costs. Multivariate data analysis is needed to integrate optical spectrometers as sensors. Therefore, a spectrometer with integrated artificial intelligence (AI) called *SmartSpectrometer* and its interface is presented. The advantages of the *SmartSpectrometer* are exemplified by its integration into a harvesting vehicle, where quality is determined by predicting sugar and acid in grapes in the field.

## 1. Introduction

The progressive digitalisation of processes in agriculture and industry requires information about plant and product parameters, preferably in real time. Results from in-line and on-line measurement sensors, instead of offline analyses in special laboratories, can be integrated directly into machines and steps of plant production to improve management decisions. Optical spectroscopy is a non-destructive measurement technique that enables high throughput at low cost and is therefore ideal as a sensor for on-line and in-line measurement.

Near infrared (NIR) spectroscopy in particular can be used to analyse the composition of e.g., liquids, solids or powders. Depending on the absorption and scattering either transmission or reflection can be applied. Relevant information can be extracted and the penetration depth ensures bulk information. Direct molecule transitions in the mid-infrared, often used for gas analysis, reveal strong absorption and thus penetration depths in the micrometer range only. For dense matter the 2nd harmonics of the molecule transitions are typically used which are prominent in the NIR range between 1000 nm and 1700 nm. The overtone and combination bands of molecules especially complex organic matter typically reveal a width in the 10 nm or more range. Thus the requirement for the analyzer will not exceed this value.

NIR spectroscopy [1,2,3] has therefore already proven its viability in numerous applications, especially in the field of food inspection and agriculture [4]. For example, ingredients of food and animal feed such as fat, carbohydrate, fibre or protein content, can be determined. In addition, there are also numerous applications in the agricultural sector, ranging from soil analysis to the determination of moisture or the degree of ripeness of the crop. This enables more sustainable agriculture with better crop yields [5].

In the last few years, near-infrared spectroscopy has made a great step forward in development. The progress in the development of micro-electromechanical systems (MEMS) has made it possible to produce miniaturised optical spectrometers of different designs in compact form at low cost and in large quantities [6]. It has become technically possible to integrate optical spectrometers into smartphones [7,8]. This means that optical spectroscopy can also be used outside of specialised laboratories [9]. For example, optical spectrometers can be used as food scanners to determine freshness [10] or ripeness [11]. However, there are also numerous other applications that have been demonstrated in recent years [12,13].

For proper operation of the spectrometer including the evaluation model, reproducibility, i.e., the stability of the wavelength scale and noise performance are very relevant parameters. Therefore, further steps are necessary to integrate optical spectrometers as intelligent sensors in the Industrial Internet of Things (IIoT). These are, for example, self-calibration and optimization, as well as multi-sensorial and multi-dimensional data evaluation [14].

This paper presents a concept of an optical spectrometer as a device with integrated data analysis and an open and standardized interface. The so-called SmartSpectrometer is designed in such a way that it can be used without further knowledge about spectroscopy. The device contains the spectrometer, calibration data and an illumination control. The analysis of the spectroscopic data is done with artificial intelligence (AI). A neural network architecture was developed for this purpose, which converges with just a few training data through multitask learning and already contains all data preprocessing. The presented neural network *AnniNet* enables a multi-sensorial data analysis and shows better results on benchmark data sets and changing measurement conditions (i.e., wavelength scale) than classical methods.

## 2. Miniaturized Optical Spectrometer

In many fields of applications, the need for compact or ultra-compact system designs arises [15]. The size must be reduced beyond the capabilities of simply downsizing existing spectrometer layouts. The first approach to miniaturize spectrometers was the use of fixed gratings and detector arrays. For ultra-compact two limiting factors arise: InGaAs detector arrays with sufficient elements—a minimum of 256 has to be considered—drive up the cost and there is a choice of either wide element sizes and lower noise or small outline. The same limitations arise for filter based systems on array detectors. Fabry–Perot filters above single element detectors could only address insufficient small wavelength intervals.

New concepts have been enabled by micro electro mechanical systems (MEMS). Tiltable mirror plates have been established decades ago. Grating structures instead the mirror have been invented since the year 2000 [16]. Furthermore piston type mirrors and in-plane integrated actuators have been realized by means of MEMS technologies.

### Grating-Based NIR Spectrometer

**Figure A1:**
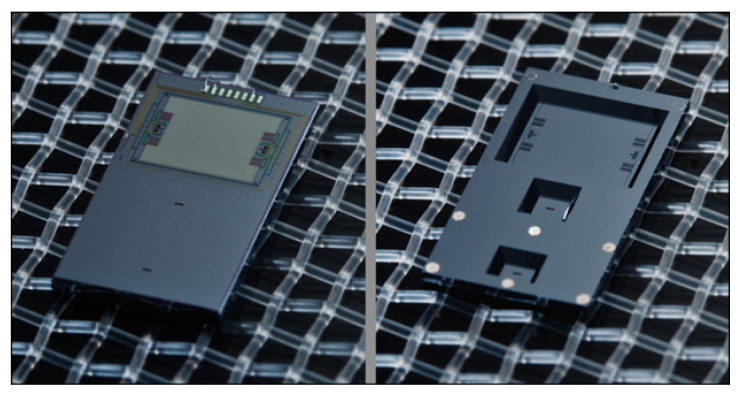
MEMS chips with tiltable grating, integrated position sensor and two slits in one device; left image shows the front side, right image shows the rear side with the cavities etched into the chip.

Deflectable optical elements like mirrors or gratings enable scanning monochromators. The optical setup is close to systems which have been used in earlier times very successfully in combination with single detectors like photo multiplier tubes. Scanning grating spectrometers using a classic Czerny–Turner W-configuration were developed for a range from 950 nm to 1900 nm, with a resolution of 10 nm and a volume of 720 cm^3^. The next step was the integration of both slits into the MEMS chip (Figure A1).

**Figure A2:**
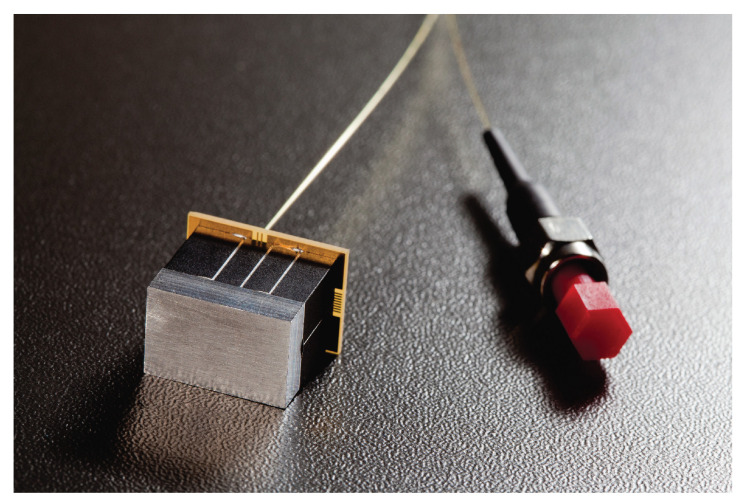
Stacked component spectrometer (“Sugar cube spectrometer”), left in the image the optical bench is depicted, fiber to a standard SMA head which indicates size on the right side of the image.

The optical system has been built based on stacking functional components. The spectrometer bench is a modified Czerny–Turner using the first diffraction order in a double-v configuration. This is necessary, as the grating cannot be tilted out of the chip plane in the rest position in this chip. The detector—an InGaAs bare die with 600 × 600 × 300 µm size—is placed in the cavity of the MEMS chip. Two off axis mirrors are needed which increases the complexity of the system. Nonetheless spectrometers with the size of a regular sugar cube (2.1 cm³) have been realized (Figure A2) which feature similar performance as the much larger scanning grating spectrometer before.

**Figure 1:**
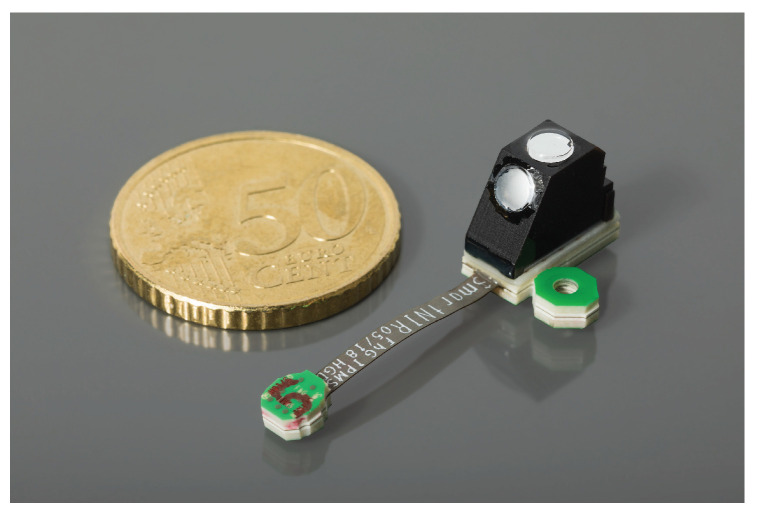
Smart NIR spectrometer, optical bench measuring 6.5 × 10 × 10 mm^3^ on a printed board with mounting bail and flat wire to connector; 50 Euro cent coin for size indication.

Another request for ultra-compact spectrometer led to the development of a further miniaturized setup. The outline has been reduced to 10 × 10 × 6 mm^3^ (Figure 1).

**Figure A3:**
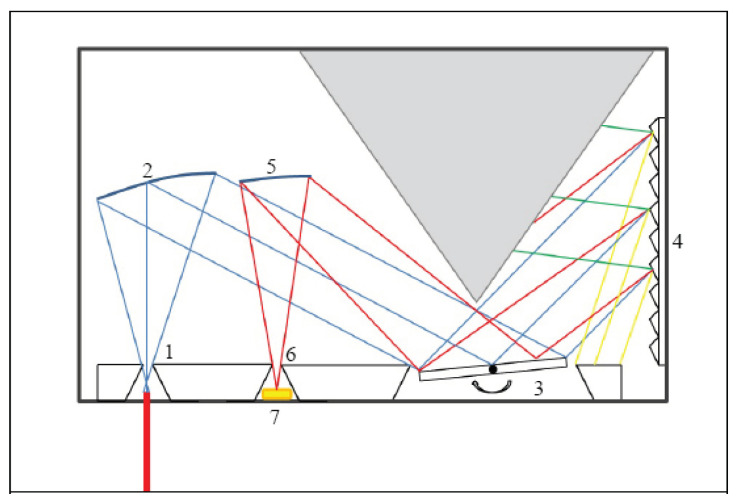
Principle of a scanning mirror micro spectrometer: 1: entrance slit, 2: collimation mirror, 3: mirror plate, 4: fixed grating, 5: refocusing mirror, 6: exit slit, 7: detector element.

Besides, the size reduction further technical optimization has been requested. The technological aspects, as well as the drive requirements, can be reduced if a spectrometer is using a simple scanner mirror which illuminated a fixed grating. Half of the deflection enables the same spectral range [17]. The grating can be adjusted very simply. A scanner mirror-based spectrometer design has been developed (Figure A3).

**Figure A4:**
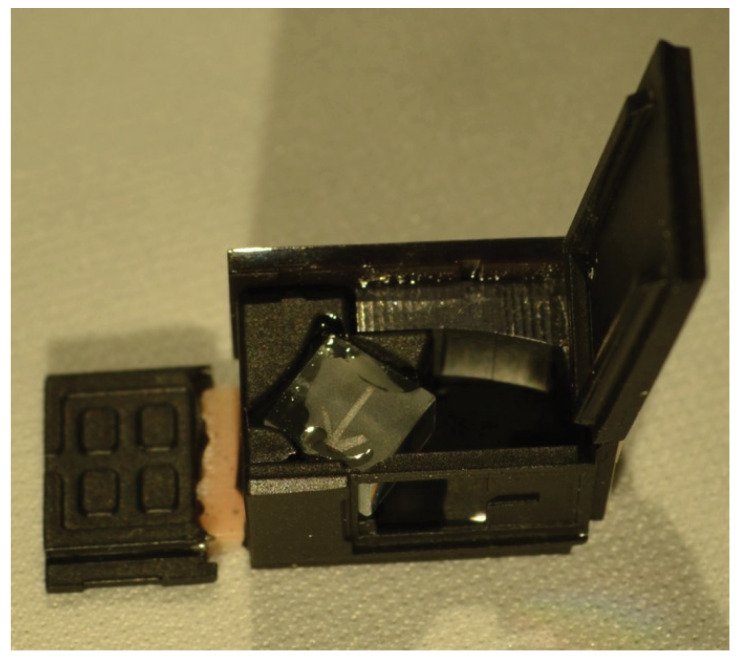
Folded optical bench for a MEMS based micro spectrometer, the fixed grating is in the left middle, both off-axis mirrors were realized in one substrate placed in the upper region of the substrate, printed board with scanner mirror and detector unit is not shown here.

Another relevant aspect for the production of spectrometer especially in large volume in the integration. Spectrometers are mostly based on off axis optical systems. Compared to camera lenses the assembly needs the three-dimensional placement of components in a system body. A new concept called “place and bend assembly” [18] uses a mostly planar substrate where the components are placed by standard 2D assembly tools. Afterwards the substrate is folded along previously designed bending [19] lines and the 3D body is generated with the optical path inside. Applying this technology a first spectrometer has been realized recently (Figure A4). Again, 10 nm resolution was achieved in a spectral range from 950 nm to 1900 nm.

Between optical system and evaluation platform is also space for some optimization. The signal-to-noise ratio (SNR) can benefit from longer integration times compared to scanning systems using resonantly driven scanner devices. The use of quasi static actuated MEMS scanner mirrors in optical systems, as used before, can be tuned to a specific wavelength and remain at this position. The signal of selected bands can be integrated using longer integration times which reduces the noise. Matching scanner mirror devices are available now but position control needs further improvement to ensure the accuracy required for spectroscopic devices. For applications like process control and monitoring this concept is very promising for future systems.

## 3. Software Architecture

### 3.1. Spectrometer as Edge Device

**Figure 2:**
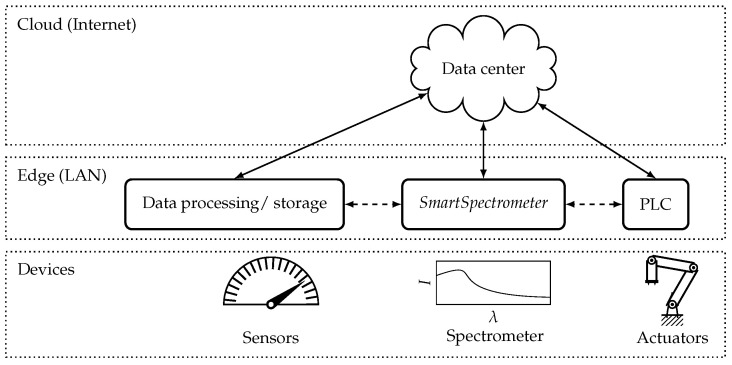
In the ongoing process of digitalization, networking is emerging at machine and factory level. Local data processing enables high availability with low latencies. In this architecture, programmable logic controller (PLC) for controlling actuators can directly access processed sensor data.

In the industrial internet of things, information from different data sources and sensors can be merged, by networking. The interpretation of spectroscopic measurement data is planned to be improved in the following by using additional information such as the temperature of a sample. The fusion of data from different sources is often associated with cloud computing. Data are stored and processed at a central location using the internet. However, data processing in the local area network (LAN) offers some advantages such as lower latency and availability. This level between the device level and the cloud is called edge. Edge devices enable horizontal connections between each device, as well as cloud connectivity (Figure 2).

*SmartSpectrometer* is designed as an edge device, of which the results are provided using Open Platform Communications Unified Architecture (OPC UA). The standardized interface via OPC UA enables the easy integration and scalability of further sensors according to the principle Plug and Work [20]. This makes it easy to add new sensors and deploy new machine learning frameworks quickly as a service within an industrial network. The vertical connection to central cloud services is possible and enables the monitoring of the entire production process.

The software is divided into different independent modules called *SensorServer* and *PredictionEngine*, which can be integrated at runtime. To implement this so called service-oriented architecture (SoA) the OPC UA implementation open62541 [21] was used.

### 3.2. Spectral Sensing Module SensorServer

In order to use an optical spectrometer as a sensor, further components such as an illumination control are necessary. The aim of the *SensorServer* is therefore to provide a uniform and manufacturer-independent interface for data acquisition and calibration routines. For this purpose, in addition to the optical spectrometers of various manufacturers, the illumination control and reference measurements are also integrated. In total, the integration of numerous optical spectrometers from the prototype to the OEM devices were realised (see Table 1).

### 3.3. Data Processing Module *PredictionEngine*

Another essential component of *SmartSpectrometer* is the spectral data analysis on sensor level (edge computing). The *PredictionEngine* is designed as a so-called aggregating OPC UA server. This means that the balanced spectral data is automatically retrieved and analysed. Furthermore, the *PredictionEngine* has the purpose of acting as an interface to various machine learning frameworks. Optionally, additional parameters can be set, e.g., by additional sensors. For example, the asynchronously recorded temperature of the sample can be set as a parameter and thus improve the spectral data analysis.

### 3.4. OPC UA Information Model

To enable communication between devices, it is necessary to define interfaces. The OPC UA standard used in present study enables a platform-independent transfer of data. In addition, the standard provides a machine-readable semantic description of the variables. The variables and methods of the SmartSpectrometer concept, including a semantic description, will be explained in the following information model. The aim of the *SmartSpectrometer* information model is to create a uniform device- and manufacturer-independent interface. The naming of all variables can be found in the Appendix.

The optical spectrometers offered by the manufacturers differ in their hardware interfaces, and some of them are spectrometer systems with integrated illumination. However, the features common to all systems are most necessary components for operation. All variables and methods of the *SensorServer* are summarised in Table A1.

An aggregating OPC UA server called *PredictionEngine* is used for the prediction. For clarity, the parameters and methods are organised in different folders (Table A2). Therefore, in addition to selecting the AI-model, the address of the *SensorServer* used must also be set in the configuration (Table A3). Also available as parameters are the index and time stamp of a measurement. Optionally, this is supplemented by the measurement data of the spectrometer (Table A4). The evaluation of the spectral data is carried out by the included AI. The *PredictionEngine* enables a uniform representation of the parameters estimated from the spectral data (Table A5). In addition, further parameters can be set asynchronously, for example supplementary information provided by external sensors. This supplementary information can improve the results of AI-models (see Table A6).

## 4. NIR Data Processing Using the Neural Network *AnniNet*

Optical spectra in the near infrared contain information about material properties through absorptions, which are caused by excititations of molecular vibrations. More precisely, polar hydrogen bonds (OH, NH, CH, etc.) in particular produce absorptions in this spectral range. This is why the technology is particularly suitable for the analysis of food and agricultural products as well as organic compounds. However, the absorptions in the spectral range of the near-infrared overlap strongly, so that the properties can only be estimated with product-specific chemometric models in a limited range of values. To complicate this, the spectral measurements are also considerably disturbed by tolerances in the manufacturing of optical spectrometers and the measurement set-up. To solve this problems, a neural network architecture is presented in the following, which includes spectral pre-processing and at the same time is tolerant to wavelength shifts of the spectral data.

The presented architecture of a convolution neural network for the analysis in the near-infrared, called *AnniNet*, is working without any additional spectral pre-processing like normalization and correction of non-linear effects. The *AnniNet* architecture is an ensemble of several networks for feature extraction (encoder) and regression. In the training, a decoder network is also used. Furthermore, in addition to the spectral features, prior knowledge like sample temperature can also be integrated.

### 4.1. Encoder Network

Wavelet-based methods are a successfully used pre-processing method in chemometrics [22]. The use of wavelet-based pre-processing can reduce noise [23] and suppress background effects [24]. Therefore, the prediction accuracy [25] and the transferability [26] of chemometric models improve. The very useful pre-processing by Norris derivatives also uses convolution with a filter function [27].

In recent years, convolutional neural networks (CNNs), especially in combination with deep learning, became the de-facto standard for complex computer vision tasks and popular for data analysis tasks in general. Neural networks have the advantage of not requiring the manual selection and implementation of feature detectors. The convolution layer can do the same as wavelet-based methods, but individual adaptations of the filters are also created during training. It is also common to use so-called pooling layers to down sample the feature map resulting from the convolutional layers. This creates a new feature map with local translation invariance, which is very important for generalisation across different spectrometers, because of a robustness against wavelength shifts.

Therefore, the encoder network is designed by convolution and pooling layers to extract information by transition the spectrum $x_{i}\in R^{Q}$ of the *i*-th sample to the feature map $M_{i}\in R^{p\times k}$. Which contains the results after convolution with k-filters reduced to the dimension p<Q by max-pooling. The use of k-different filters is a significant added value compared to classical chemometrics, which is usually based on the use of a single filter function. Furthermore, the filter parameters are learned from the data and do not have to be determined by experts.

#### Decoder Network

**Figure 3:**
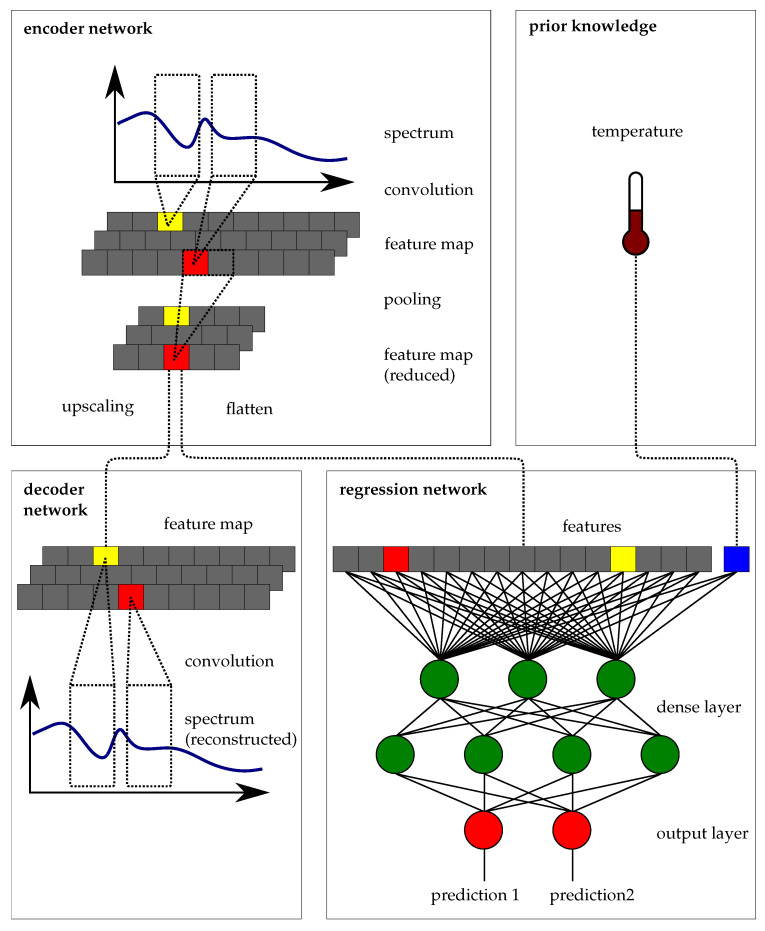
*AnniNet* is essentially made up of three components. An encoder network, which extracts the features through a convolutional layer and then condenses them. A decoder network, which is used in training to improve feature extraction. And the evaluation of the spectral information is done in a regression network of dense layers, here the non-linear and overlapping spectral features can be evaluated. In addition, prior knowledge, for example the sample temperature, can be added to the regression network.

Typically, the data sets in optical spectroscopy are much smaller than in image processing, where neural networks have already had great success. To improve the training of the encoder network, an additional decoder network was used. This results in an so called autoencoder architecture. Unsupervised learning without labels has been used for training of autoencoders, which is a major advantage since labeled spectral data can be very expensive to produce [28]. More precisely, autoencoder architectures are used to reconstruct the input, of which the size is compressed after transforming it to internal hidden layers. Therefore, the hidden layers represent the features of the input with reduced dimensionality. The decoder network of *AnniNet* is designed by convolution and up sampling layers to reconstruct the spectrum ${\hat{x}}_{i}\in R^{Q}$ from of the *i*-th sample from the feature map $M_{i}\in R^{p\times k}$. The decoder network thus supports the learning of essential features of the spectrum without the need for additional labels (Figure 3).

### 4.2. Regression Network

Spectral data contain information through absorptions that lead to local intensity changes. Fully connected (dense) layers are able to learn these complex and superimposed relationships. Due to the exponential relationship between absorption and constituents (Beer–Lambert law), a dense layer with Scaled Exponential Linear Unit (SELU)

(1) $$f_{\mathrm{SELU}}\left(x\right)=\left\{\begin{array}{cc} \lambda \alpha (e^{x}-1), & \mathrm{if}\;x<0\\ \lambda x, & \mathrm{otherwise} \end{array}\right.$$

as activation function is applied first. This is followed by a layer with softplus

(2) $$f_{\mathrm{softplus}}\left(x\right)=\ln\left(1+e^{x}\right)$$

activation function.

Overall, the regression network is designed by fully connected layers to predict the target parameters ${\hat{y}}_{i}\in R^{C}$ of the *i*-th sample from the feature map $M_{i}\in R^{p\times k}$. Optionally, the feature map M can be extended to include prior knowledge like sample temperature.

### 4.3. Multi-Task-Learning

In multi-task learning, all target parameters Y and spectra X of the training data set are used in parallel. The sum of the root mean square errors

(3) $${\mathrm{RMSE}}_{\mathrm{reconstruction}}=\sum_{i}\sqrt{\frac{\sum_{j=1}^{Q}{\left({\hat{x}}_{i,j}-x_{i,j}\right)}^{2}}{Q}}$$

and

(4) $${\mathrm{RMSE}}_{\mathrm{regresssion}}=\sum_{i}\sqrt{\frac{\sum_{j=1}^{C}{\left({\hat{y}}_{i,j}-y_{i,j}\right)}^{2}}{C}}$$

is used to optimise the reconstruction and regression. In addition, dropout is included in training to reduce overfitting. Using expected perturbations like wavelengths shifts or variations of the baseline is a efficient method to develop robust calibration models [29]. Therefore, data augmentation using baseline variations and wavelengths shifts was also used.

### 4.4. Prior Knowledge

Near-infrared spectroscopy mainly analyses the vibrational excitations of molecules. But the vibrational states of molecules are subjected to external influences like temperature and pressure. For this reason, the shape of particular absorption bands in the near infrared is temperature and pressure dependent.

Therefore, *AnniNet* is designed to include additional knowledge of an sample. This additional knowledge is provided as further input to the regression network described before. The additional input can also be provided multiple times in different layers of the fully linked regression network (Figure 3).

### 4.5. *AnniNet* Evaluation

A wavelength shift is a serious problem in transfer of chemometric models and involves typically a lot of effort. Typically, special instrument transfer methods are required [29]. Due to the small sizes, wavelength shifts caused by manufacturing tolerances and thermal expansion are significantly increased in miniaturized spectrometers.

The architecture of *AnniNet* has an increased robustness against wavelength shifts due to locally translation invariant feature extraction. To show this, the *AnniNet* architecture is compared with the frequently used Partial Least Squares Regression (PLSR) and the Least Squares Support Vector Regression (LSSVR) with an RBF kernel. The LSSVR is a non-linear regression

(5) $$y_{i}=\sum_{i=1}^{n}\alpha_{i}K\left(x,x_{i}\right)+b$$

using the RBF kernel function *K*. The regularization parameter γ and the bandwidth of the RBF kernel σ are chosen as described in [30].

The cargill corn data set [31] is a widely used benchmark data set for instrument transfer. It contains 80 corn samples measured using 3 different near infrared spectrometers encoded as m5, mp5, and mp6 with different properties and overlapping wavelength regions. Each spectrum contains Q=700 spectral bands in the wavelength range of 1100–2498 nm with a resolution of 2 nm. The training is based on 60 samples randomly selected from the parent instrument. The validation was carried out based on the prediction of the remaining 20 samples on the respective child instrument.The results are shown in Table 2.

The *AnniNet* results are better in predicting moisture content than the PLSR and LSSVR, which are used for comparison. This is even more the case for prediction on unknown instruments. In the case of the similar instruments mp5 and mp6, there are no significant differences to known procedures. The prediction on the slightly different spectrometer m5, are clearly better than results of the comparison methods, but by using labelled and unlabelled data of the target spectrometer, partly better results can be achieved [32].

A benchmark for influence of temperature on vibrational spectra is a data set of mixtures of isopropanol, ethanol, and water [33]. The spectra are recorded at different temperatures of the samples from 30 °C to 70 °C. A division into training and test samples similar to [33] and spectra of all temperatures have been used. In an additional experient, a wavelength shift of one spectral band was applied. This corresponds to a shift of 0.97 nm, which is quite realistic. The results are shown in Table 3.

The spectral data were pre-processed by standard normal variate (SNV) before applying PLSR and LSSVR. An optimal number of n = 7 components was determined for the PLSR. The RMSEs in the non-disturbed case are comparable to results achieved in literature [34]. Adding temperature as a feature to the PLSR model has no positive effect [35]. The methods to achieve robustness of the models to changes in temperature [36] are often also used for instrument transfer. After a wavelength shift, the results are significantly worse. For the non-perturbed case, the RMSE of *AnniNet* is comparable to the nonlinear LSSVR regression. However, the RMSE of *AnniNet* is almost unaffected by a wavelength shift. As expected from the locally invariant feature extraction, *AnniNet* is robust against small wavelength shifts.

## 5. Application of Embedded NIR-Spectroscopy on a Grape Harvester

Producing high quality wine by combining the best parcels of fruit from one or several grapevine varieties is by far the most important goal of wine cooperatives in Germany. Often they have more than one hundred cooperative members, which have several parcels of one variety. The uniformity of different batches of fruit in addition to its sensory attributes is a key aspect for the produced wine quality. In viticulture, it is much easier to transfer uniform batches of fruit into a wine of desired style, flavor and aroma than batches that might cover different ranges of quality [37]. So far, the decision of combining different batches of fruit can be made only after measuring the gained must of the delivered batches in the wine cooperative. Sometimes even several batches are delivered together what makes it even more difficult to actively combine batches of different quality, since they are already delivered as a mixed sample.

**Figure 4:**
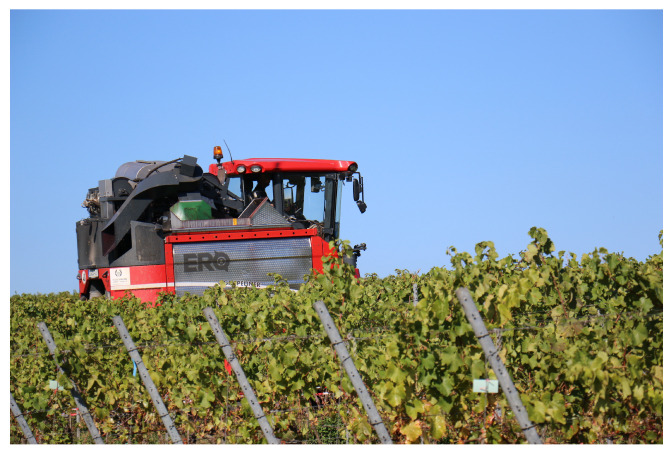
Grape harvester during harvest in the vineyard.

Integrating a compact optical spectrometer as an embedded device with integrated data processing into a grape harvester (Figure 4) can provide a solution for the timely determination of the quality attributes of the grapes, such as sugar and acidity during field harvest. In combination with data transmission to the wine cooperative’s batch planning software, this would improve efficiency of the planning of the grape receiving department and the winemakers decision making enormously. The recorded results would be used to generate a yield and quality mapping, which could further promote management decisions in the future through a better understanding of the spatial variation of fruit quality within single parcels.

The *SmartSpectrometer* concept presented is well suited for use on a grape harvester. The estimation of quality attributes by a low-cost miniature spectrometer at varying sample temperature is therefore evaluated in the following.

### 5.1. Data Set

Must and grape clusters from the grapevine varieties Pinot Noir and Dornfelder (*Vitis vinifera*, L.) were collected from eight vineyards in Rhineland palatinate (Germany), among which six are located in Wollmesheim (49°11′00.3″ N 8°05′58.0″ E), one in Mühlhofen (49°06′41.4″ N 8°05′28.7″ E) and one in Deidesheim (49°24′55.4″ N 8°11′35.9″ E). All rows were north - south orientated, with the exception of those in Deidesheim (east–west orientation). The greening was well-tended. Samples were collected from 18 September 2020 to 30 September 2020 for Pinot Noir and from 14 September 2020 to 15 September 2020 for Dornfelder. Four must samples per plot were taken from the grape harvester’s tank during harvest. Grape clusters from two or four vines were collected one week post harvest and stored at 5 °C before manually juiced with a hydraulic press (Hydropresse Edelstahl 40 L, Speidel, Ofterdingen, Germany). All samples were stored in dark at 5 °C before measurement. The value range of the data set is described in Table 4.

#### 5.1.1. Reference Analysis

Samples were filled in 50 mL falcon tubes and centrifuged at 13,500 rpm (Sigma 6-16KS, Sigma, Kawasaki, Japan) for 5 min and filtered with 100 µm sieves. For spectral measurement the supernatants were filled in syringes with a 8 µm syringe filter in front. For reference measurement, 1 ml of supernatant was 1:3 diluted with filtered water (0.2 nm filters) and centrifuged again with 13,400 rpm (Minispin Eppendorf, Hamburg, Germany). The contents of glucose, fructose, tartaric and malic acid were determined with high performance liquid chromatography (Agilent 12900 Infinity II, Agilent technologies Inc., Santa Clara, CA, USA) consisting of a multisampler (G7167B), a binary pump (G7120A), a Rezex™ ROA-Organic Acid H^+^ column (300 × 7.8 mm, 8 µm) protected by a Security Guard™ Carbo-H^+^ column (Phenomenex Inc., Torrance, USA) kept in a column oven (G7116B) at 60°C, a diode array detector (G7117B), and a refractive index detector (G1362A) kept at 50 °C. Each sample was analyzed for 16.5 min under a mobile phase of 4 mM sulfuric acid solution with a flow rate of 0.6 mL/min. The injection volume was 5 µL. A dilution series ranging from 15 g/L to 90 g/L of sugars and 0.25 g/L and 15 g/L of acids was used as standard. Data were analyzed with Agilent OpenLab CDS Chemstation software (Agilent Technologies Inc, Santa Clara, CA, USA).

#### 5.1.2. Spectroscopic Measurements

The prediction of sugar and acidity from near-infrared spectra of grape juice is subject to several challenges. First, the near infrared spectrum is strongly affected by the absorption of water, especially at wavelengths above 1400 nm. Furthermore, ranges of the near-infrared spectrum above 1700 nm can only be detected by expensive and cooled spectrometers. Therefore, the use of a low-cost MEMS-FPI spectrometer (NIRONE1.4, Spectral Engines) in the measuring range of 1100–1350 nm was chosen. Because the absorptions in this spectral range are only weak, a flow cell (FIA-1000-Z-SS, Ocean Optics) with an optical path length of 10 mm was used. The only preparation of the grape juice consisted of mechanical filtering, all particles larger than 20 μm were removed.

The selected spectral range covers the known absorptions by excitation of the second harmonic oscillation of the CH_x_ groups, which are also components of acid and sugar. Furthermore, absorptions by OH groups are known in this spectral range, which vary depending on the H-bond. Absorption by the OH groups is particularly problematic because the structure-breaking effect makes the spectral changes caused by the addition of solutions in water very similar to a change in temperature [38].

**Figure 5:**
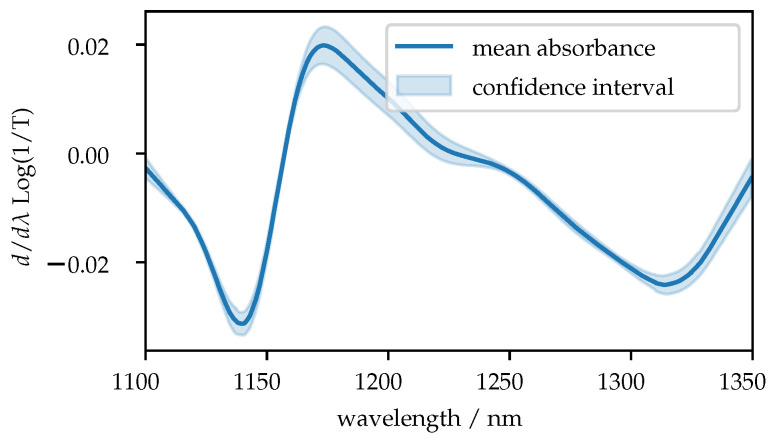
Shown is the first derivative of the measured absorbance of all samples at a sample temperature of 20 °C. For a better representation, a SNV was carried out. The confidence interval shows the differences between samples with different sugar and acid contents.

In order to take the influence of temperature into account, the flow cell was regulated to 20 °C and 30 °C in two measurement series. The near-infrared spectra were recorded with a low-cost MEMS-FPI spectrometer. The spectra are shown in Figure 5, for better visibility a normalization using Standard Normal Variate (SNV) was performed and the first derivative was applied.

### 5.2. Results and Discussion

To achieve the best possible applicability and to extend the range of parameters, a cross-product model of the two red wine varieties Pinot Noir and Dornfelder was trained. The *AnniNet* result is compared with the linear PLSR and non-linear LSSVR. Training and comparison methods are the same as in the previous section. Spectral data pre-processing for *AnniNet* is not applied. The best results for the PLSR and LSSVR were obtained after filtering by Savitzky-Golay (7, 2), generating the first derivative and normalising by using Standard Normal Variate (SNV). An optimal number of 5 components was determined for the PLSR. For training, 75% randomly selected samples were used, 25% of the samples were used for testing. The results of the prediction of the test samples are shown in Table 5.

The best estimation of the quality parameters was done by *AnniNet*, there is a clear gap to the linear and non-linear comparison methods PLSR and LSSVR. This is in agreement with the previous results of the benchmark data sets. Under the influence of external disturbances such as wavelength shift or instrument transfer, *AnniNet* also achieved significantly better results. The reason of these differences is probably a robust feature extraction. The use of the sample temperature as additional knowledge improves the prediction of sugar content.

In addition, an experiment was performed to determine the impact of specific regions of the spectra. For this purpose, a attribution map was created. Parts of the spectrum are masked out window by window and the prediction error was determined. If the masking of a spectral range leads to high errors in the prediction, the spectral range can be considered important for the prediction.

**Figure 6:**
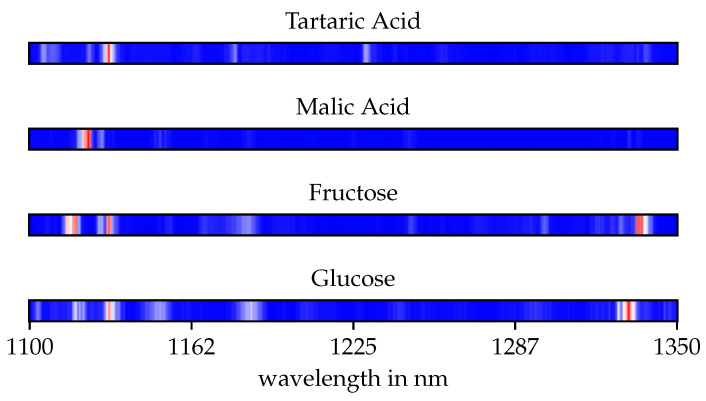
The attribution maps show the regression error caused by mask-ing out individual spectral bands. Areas marked in red have a high importance for the regression of the corresponding target parameter.

The calculated attribution map (see Figure 6) shows that the network uses different regions of the spectra depending on the parameters. The specific spectral ranges are narrow and sparse, this is a result after applying dropout in training. Dropout is a regularisation process that reduces overfitting and tends to sparsity. Furthermore, it can be seen in the attribution map that the estimation of both acids mainly depends on the range between 1100 nm and 1160 nm. For the determination of sugars, the attribution map shows additional importance of the range between 1300 nm and 1350 nm.

## 6. Conclusions

The presented *SmartSpectrometer* is a concept of interfaces and algorithms for an optical spectrometer as an embedded sensor system with integrated data analysis at sensor level (edge device).

Using the *SmartSpectrometer* concept, a spectrometer can easily be replaced by another one, because all hardware-specific properties are stored in the device. In addition, all spectral measurements and properties like calibration data can be accessed in the same way. An OPC UA server is used for this purpose, the information model was described as *SensorServer*.

The data analysis is realised as an independent software module *PredictionEngine* and offers the results of all predictions using an OPC UA interface. The information model also offers the possibility to integrate additional knowledge or correction coefficients.

The heart of the *PredictionEngine* is an integrated AI unit. The neural network *AnniNet* developed for this purpose has achieved significantly higher prediction accuracies on published data sets than the frequently used PLSR. Non-linear regression models such as LSSVR can achieve comparable results, but even small shifts in wavelengths lead to a drop in prediction accuracy. *AnniNet* has a locally translation invariant feature extraction using pooling and is therefore particularly suitable for MEMS-based miniature spectrometers with increased uncertainty in the wavelength calibration.

The combination of miniaturised spectrometer technology, a spectrometer-independent network interface and a neural network that requires no instrument transfer enables the use of NIR spectroscopy for smart sensor IIoT applications. The edge computing architecture with local data processing is particularly suitable for use on machines without a permanent internet connection. In an exemplary real application on a grape harvester, the presented data analysis algorithm was able to achieve very good results with a low-cost spectrometer.

## Figures and Tables

**Table 1:** Summary of the optical spectrometers included in the *SensorServer* Software.

Device	Manufacturer	Technology	Detector	Range	Inteface
Sugar Cube	Fraunhofer IPMS	scanning grating	InGaAs diode (uncooled)	950–1900 nm	UART/SPI
NIRONE	Spectral Engines	MEMS Fabry-Pérot interferometer	InGaAs diode (uncooled)	1100–2500 nm (type specific)	I${}^{2}$C/USB
NeoSpectra	Si-Ware	MEMS FTIR	InGaAs diode (uncooled)	1300–2500 nm	SPI
Flame-NIR	Ocean Optics	Grating	InGaAs diode array (uncooled)	900–1700 nm	USB
NIRQuest	Ocean Optics	Grating	InGaAs diode array (cooled)	900–1700 nm	USB
Rock XNIR	Ibsen Photonics	Grating	InGaAs diode array (cooled)	1100–2200 nm	USB
DLP NIRscan Nano	Texas Instruments	MEMS DLP	InGaAs diode (uncooled)	900–1700 nm	USB

**Table 2:** RMSE of the prediction of moisture content of the Cargill data set. For the training, only training samples of the spectrometer named as parent were used. The evaluation was done using the test samples on the instruments named (child instruments). The *AnniNet* shows very good results in predicting new samples on new devices compared to the Partial Least Squares Regression (PLSR) and the Least Squares Support Vector Regression (LSSVR).

Model	Parent	m5	mp5	mp6
*AnniNet*	m5	0.13	0.63	0.59
	mp5	0.33	0.20	0.21
	mp6	0.21	0.21	0.21
LSSVR	m5	0.18	1.68	2.06
	mp5	0.90	0.24	0.79
	mp6	1.03	0.57	0.19
PLSR	m5	0.19	1.93	2.16
	mp5	0.86	0.24	0.42
	mp6	0.32	0.29	0.28

**Table 3:** RMSE of the prediction of the different mixture proportions after using *AnniNet*, partial least squares regression (PLSR) and the least squares support vector regression (LSSVR). Results marked with * after a perturbation by a shift of one spectral band (0.97 nm) compared to the training. *AnniNet* shows stable results even with wavelength shifts.

Target	*AnniNet*	PLSR	LSSVR	*AnniNet* *	PLSR *	LSSVR *
Ethanol	0.0069	0.0124	0.0065	0.0086	0.2371	0.2406
Isopropanol	0.0060	0.0112	0.0066	0.0075	0.2459	0.2473
Water	0.0039	0.0078	0.0040	0.0037	0.0182	0.0202

**Table 4:** *SmartSpectrometer* data set with samples of different red wine varieties.

Varieties	Samples	Malic Acid (g/L)	Tartaric Acid (g/L)	Fructose (g/L)	Glucose (g/L)
Dornfelder	28	0.88–3.31	3.15–4.48	42.14–93.46	49.35–106.42
Pinot Noir	32	2.11–5.89	2.95–6.68	66.51–135.55	82.99–114.17

**Table 5:** Prediction error of grape quality and ripeness parameter. The RMSE is given in g/L and the R^2^ score is shown in brackets. *AnniNet* marked with * does not use temperature information as prior knowledge.

Model	Data Set	Malic Acid	Tartaric Acid	Fructose	Glucose
*AnniNet*	20 °C	0.33 (0.91)	0.32 (0.89)	6.27 (0.92)	5.46 (0.91)
	30 °C	0.43 (0.86)	0.33 (0.89)	5.99 (0.93)	4.24 (0.95)
	20 °C + 30 °C	0.4 (0.87)	0.35 (0.88)	6.46 (0.91)	4.86 (0.93)
*AnniNet* *	20 °C + 30 °C	0.4 (0.87)	0.35 (0.88)	7.27 (0.89)	5.5 (0.91)
LSSVR	20 °C	0.45 (0.83)	0.54 (0.7)	16.0 (0.48)	15.64 (0.28)
	30 °C	0.51 (0.79)	0.55 (0.7)	12.15 (0.69)	11.75 (0.58)
	20 °C + 30 °C	0.48 (0.81)	0.56 (0.68)	12.51 (0.68)	12.24 (0.55)
PLSR	20 °C	0.55 (0.74)	0.5 (0.74)	17.7 (0.36)	16.47 (0.2)
	30 °C	0.61 (0.71)	0.54 (0.71)	15.07 (0.53)	13.47 (0.45)
	20 °C + 30 °C	0.59 (0.72)	0.55 (0.7)	15.8 (0.48)	14.78 (0.35)

## Appendix A

**Table A1:** OPC-UA Information Model of the SensorServer.

Attribute	Value				
BrowseName	PointSpectrometer				
IsAbstract	False				
**Reference**	**NodeClass**	**BrowseName**	**DataType**	**TypeDefinition**	**ModdelingRule**
HasComponent	Variable	SensorName	String	BaseDataVariableType	Mandatory
HasComponent	Variable	SensorID	String	BaseDataVariableType	Mandatory
HasComponent	Variable	SensorFirmwareVersion	String	BaseDataVariableType	Mandatory
HasComponent	Variable	IntegrationTime	String	BaseDataVariableType	Mandatory
HasComponent	Variable	MinIntegrationTime	String	BaseDataVariableType	Mandatory
HasComponent	Variable	MaxIntegrationTime	String	BaseDataVariableType	Mandatory
HasComponent	Method	AcquireWhiteSpectrum			Mandatory
HasComponent	Method	AcquireBlackSpectrum			Mandatory
HasComponent	Method	Balancing			Optional
HasComponent	Method	AcquireReferenceSpectrum			Optional
HasComponent	Method	DeleteReferences			Optional
HasComponent	Method	Calibration			Optional
HasComponent	Method	AcquireRawSpectrum			Mandatory
HasComponent	Method	AcquireSpectrum			Mandatory
HasComponent	Method	StartContiniousMode			Mandatory
HasComponent	Method	StopContiniousMode			Mandatory
HasComponent	Variable	CycleTime			Mandatory
HasComponent	Variable	Spectrum			Mandatory
HasComponent	Variable	RawSpectrum			Mandatory
HasComponent	Variable	Wavelengths			Mandatory

**Table A2:** OPC-UA Information Model of the PredictionEngine.

Attribute	Value				
BrowseName	Predictor				
IsAbstract	False				
**Reference**	**NodeClass**	**BrowseName**	**DataType**	**TypeDefinition**	**ModdelingRule**
HasComponent	Method	Update			Mandatory
Organizes	Object	Config		FolderType	Mandatory
Organizes	Object	Measurement		FolderType	Mandatory
Organizes	Object	Prediction		FolderType	Mandatory
Organizes	Object	SupplementaryData		FolderType	Optional

**Table A3:** OPC-UA Information Model of the PredictionEngine configuration.

Attribute	Value				
BrowseName	Config				
IsAbstract	False				
**Reference**	**NodeClass**	**BrowseName**	**DataType**	**TypeDefinition**	**ModdelingRule**
HasComponent	Variable	SensorServerURL	String	BaseDataVariableType	Mandatory
HasComponent	Variable	ModelURL	String	BaseDataVariableType	Mandatory
HasComponent	Method	StartContiniousMode			Optional
HasComponent	Method	StopContiniousMode			Optional
HasComponent	Variable	CycleTime			Optional

**Table A4:** OPC-UA Information Model of the PredictionEngine’s measurements.

Attribute	Value				
BrowseName	Measurement				
IsAbstract	False				
**Reference**	**NodeClass**	**BrowseName**	**DataType**	**TypeDefinition**	**ModdelingRule**
HasComponent	Variable	Index	String	BaseDataVariableType	Mandatory
HasComponent	Variable	Timestamp	String	BaseDataVariableType	Mandatory
HasComponent	Variable	Intensities	String	BaseDataVariableType	Optional
HasComponent	Variable	Wavelengths	String	BaseDataVariableType	Optional

**Table A5:** OPC-UA Information Model of the PredictionEngine’s predictions.

Attribute	Value				
BrowseName	Prediction				
IsAbstract	False				
**Reference**	**NodeClass**	**BrowseName**	**DataType**	**TypeDefinition**	**ModdelingRule**
HasComponent	Variable	<Parameter 1>	String	BaseDataVariableType	Mandatory
HasComponent	Variable	<Parameter 2>	String	BaseDataVariableType	Mandatory
			⋮
HasComponent	Variable	<Parameter n>	String	BaseDataVariableType	Mandatory

**Table A6:** OPC-UA Information Model of the PredictionEngine’s supplementary data.

Attribute	Value				
BrowseName	Supplementary				
IsAbstract	False				
**Reference**	**NodeClass**	**BrowseName**	**DataType**	**TypeDefinition**	**ModdelingRule**
HasComponent	Variable	<Parameter 1>	String	BaseDataVariableType	Optional
HasComponent	Variable	<Parameter 2>	String	BaseDataVariableType	Optional
			⋮
HasComponent	Variable	<Parameter n>	String	BaseDataVariableType	Optional

## References

[1] Siesler H.W., Ozaki Y., Kawata S., Heise H.M. (2008). Near-Infrared Spectroscopy: Principles, Instruments, Applications.

[2] Pasquini C. (2018). Near infrared spectroscopy: A mature analytical technique with new perspectives—A review. Anal. Chim. Acta.

[3] Williams P., Manley M., Antoniszyn J. (2019). Near Infrared Technology: Getting the Best Out of Light.

[4] Cortés V., Blasco J., Aleixos N., Cubero S., Talens P. (2019). Monitoring strategies for quality control of agricultural products using visible and near-infrared spectroscopy: A review. Trends Food Sci. Technol..

[5] Osborne B.G. (2000). Near-Infrared Spectroscopy in Food Analysis. Encyclopedia of Analytical Chemistry.

[6] Grüger H., Knobbe J., Pügner T., Reinig P., Meyer S. (2018). Bringing NIR spectrometers into mobile phones. MOEMS Miniaturized Syst. XVII.

[7] McGonigle A.J.S., Wilkes T.C., Pering T.D., Willmott J.R., Cook J.M., Mims F.M., Parisi A.V. (2018). Smartphone spectrometers. Sensors.

[8] Rateni G., Dario P., Cavallo F. (2017). Smartphone-based food diagnostic technologies: A review. Sensors.

[9] Crocombe R.A. (2018). Handheld spectrometers in 2018 and beyond: MOEMS, photonics, and smartphones. MOEMS Miniaturized Syst. XVII.

[10] Goisser S., Krause J., Fernandes M., Mempel H. (2019). Determination of tomato quality attributes using portable NIR-sensors. OCM 2019—Optical Characterization of Materials: Conference Proceedings.

[11] Das A.J., Wahi A., Kothari I., Raskar R. (2016). Ultra-portable, wireless smartphone spectrometer for rapid, non-destructive testing of fruit ripeness. Sci. Rep..

[12] Hintschich S., Pügner T., Knobbe J., Schröder J., Reinig P., Grüger H., Schenk H. (2014). MEMS-based miniature near-infrared spectrometer for application in environmental and food monitoring. Int. J. Smart Sens. Intell. Syst..

[13] Crocombe R.A., Leary P.E., Kammrath B.W. (2021). Portable Spectroscopy and Spectrometry, Applications.

[14] Eifert T., Eisen K., Maiwald M., Herwig C. (2020). Current and future requirements to industrial analytical infrastructure—Part 2: Smart sensors. Anal. Bioanal. Chem..

[15] Pügner T., Knobbe J., Grüger H. (2016). Near-Infrared Grating Spectrometer for Mobile Phone Applications. Appl. Spectrosc..

[16] Grueger H., Wolter A., Schuster T., Schenk H., Lakner H.K., Courtois B., Khounsary A.M., Behringer U.F.W., Uttamchandani D.G. (2003). Realization of a spectrometer with micromachined scanning grating. MEMS/MOEMS: Advances in Photonic Communications, Sensing, Metrology, Packaging and Assembly.

[17] Grüger H., Knobbe J., Pügner T., Piyawattanametha W., Park Y.H., Zappe H. (2019). MEMS based NIR spectrometer with extended spectral range. MOEMS and Miniaturized Systems XVIII.

[18] Grüger H., Knobbe J., Sabiha M.H., Piyawattanametha W., Park Y.H., Zappe H. (2019). Investigation of mechanical and optical properties of 3D printed materials serving as substrate for place and bend assembly. MOEMS and Miniaturized Systems XVIII.

[19] Grüger H., Knobbe J., Pügner T., Leuckefeld M., Reinig P., Meyer S. (2017). Concept for a new approach to realize complex optical systems in high volume. Optifab 2017.

[20] Profanter S., Tekat A., Dorofeev K., Rickert M., Knoll A. OPC UA versus ROS, DDS, and MQTT: Performance Evaluation of Industry 4.0 Protocols. Proceedings of the 2019 IEEE International Conference on Industrial Technology (ICIT).

[21] open62541: An Open Source Implementation of OPC UA. https://open62541.org/.

[22] Singh C.B., Choudhary R., Jayas D.S., Paliwal J. (2010). Wavelet analysis of signals in agriculture and food quality inspection. Food Bioprocess Technol..

[23] Mittermayr C.R., Nikolov S.G., Hutter H., Grasserbauer M. (1996). Wavelet denoising of Gaussian peaks: A comparative study. Chemom. Intell. Lab. Syst..

[24] Tan H., Sum S.T., Brown S.D. (2002). Improvement of a standard-free method for near-infrared calibration transfer. Appl. Spectrosc..

[25] Fu X., Yan G., Chen B., Li H. (2005). Application of wavelet transforms to improve prediction precision of near infrared spectra. J. Food Eng..

[26] Trygg J., Wold S. (1998). PLS regression on wavelet compressed NIR spectra. Chemom. Intell. Lab. Syst..

[27] Hopkins D. (2001). What is a Norris derivative?. NIR News.

[28] Liu T., Li Z., Yu C., Qin Y. (2017). NIRS feature extraction based on deep auto-encoder neural network. Infrared Phys. Technol..

[29] Workman J.J. (2018). A Review of Calibration Transfer Practices and Instrument Differences in Spectroscopy. Appl. Spectrosc..

[30] Suykens J.A.K., Lukas L., Vandewalle J. Sparse approximation using least squares support vector machines. Proceedings of the 2000 IEEE International Symposium on Circuits and Systems (ISCAS).

[31] NIR of Corn Samples for Standardization Benchmarking. http://www.eigenvector.com/data/Corn/.

[32] Poerio D.V., Brown S.D. (2018). Dual-Domain Calibration Transfer Using Orthogonal Projection. Appl. Spectrosc..

[33] Wülfert F., Kok W.T., Smilde A.K. (1998). Influence of Temperature on Vibrational Spectra and Consequences for the Predictive Ability of Multivariate Models. Anal. Chem..

[34] Thissen U., Pepers M., Üstün B., Melssen W.J., Buydens L.M. (2004). Comparing support vector machines to PLS for spectral regression applications. Chemom. Intell. Lab. Syst..

[35] Wülfert F., Kok W.T., De Noord O.E., Smilde A.K. (2000). Linear techniques to correct for temperature-induced spectral variation in multivariate calibration. Chemom. Intell. Lab. Syst..

[36] Hageman J.A., Westerhuis J.A., Smilde A.K. (2005). Temperature robust multivariate calibration: An overview of methods for dealing with temperature influences on near infrared spectra. J. Near Infrared Spectrosc..

[37] Bramley R., Reynolds A.G. (2010). 12-Precision Viticulture: Managing vineyard variability for improved quality outcomes. Managing Wine Quality.

[38] Giangiacomo R. (2006). Study of water–sugar interactions at increasing sugar concentration by NIR spectroscopy. Food Chem..

